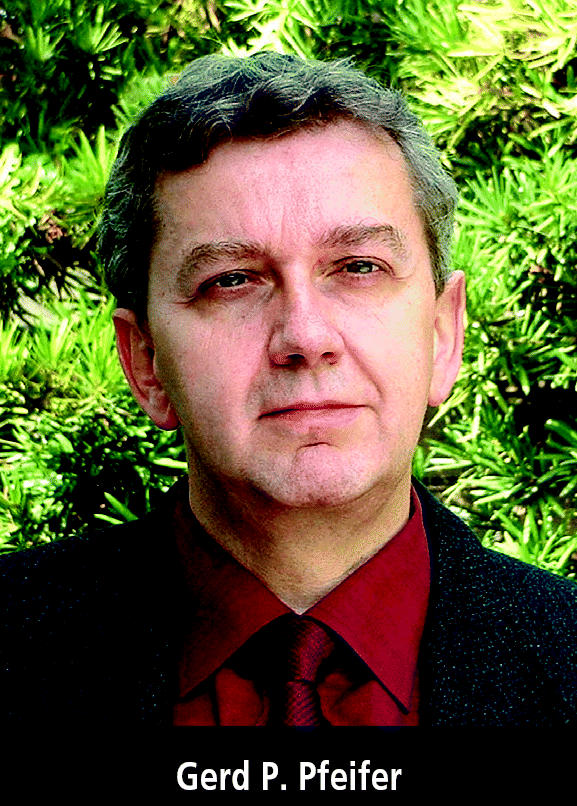# Merit Award Winners

**Published:** 2004-10

## Abstract

The NIEHS is pleased to announce that Raymond F. Burk, a professor of pathology and director of the Clinical Nutrition Research Unit at Vanderbilt University Medical Center, and Gerd P. Pfeifer, a professor and chair of the Division of Biology at the City of Hope Beckman Research Institute, have each received an award under the Method to Extend Research in Time (MERIT) Award Program. MERIT awards are offered to investigators who have demonstrated superior skill and outstanding productivity during the course of their previous research endeavors. MERIT awards relieve investigators from writing frequent renewal applications by providing the opportunity to gain up to 10 years of support in two segments.

Burk’s research has centered on the characterization of the biological function of selenium, an essential human nutrient found in virtually all tissues, and the determination of selenoproteins responsible for that function. Early work on this grant identified and characterized selenoprotein-P (Se-P), a plasma protein made primarily in the liver that carries selenium to the brain and testis. Mice lacking the protein have male infertility and brain injury; conversely, a high-selenium diet prevents brain injury.

Toxicity studies by this lab demonstrated that selenium deficiency upregulates enzymes important to the detoxification of some compounds, while the same deficiency increases the toxicity of other compounds. Burk has shown that cirrhosis is associated with low plasma Se-P and has collaborated in studies showing that selenium deficiency is not necessary for the development of colorectal adenomas. Current studies are evaluating the possibility that the Se-P delivery system to the brain can be impaired and lead to human neurodegeneration. Burk is also investigating the mechanism of transport of selenium via Se-P from mother to fetus.

As a clinical physician and former director of the Clinical Nutrition Research Unit, who has received awards for his work from the American Institute of Nutrition, Burk is in a unique position to translate research conducted in animals to human clinical research.

Pfeifer’s research has contributed greatly to our understanding of the molecular mechanisms associated with skin carcinogenesis in response to ultraviolet (UV) light injury and to the study of site specificity of UV photoproducts. He demonstrated that a different pattern of damage is observed when cells (as opposed to naked DNA) are irradiated with UV light, and he characterized the methylation and nucleosome locations of the p53 genes.

With colleague Gerald P. Holmquist, Pfeifer applied ligation-mediated polymerase chain reaction to the study of DNA damage at the nucleotide level. This allowed the critical observation that differences in mutation frequency depend upon the efficiency of DNA repair rather than lesion induction alone. The Pfeifer lab demonstrated an important role of the DNA base 5-methylcytosine in UV damage formation and mutagenesis.

Pfeifer proposes to characterize DNA-damaging and mutagenic properties of UVA irradiation, a component of the solar spectrum that has been linked to melanoma. He then will attempt to demonstrate a molecular link between sunlight exposure and melanoma. Studies will determine the *in vivo* roles of DNA polymerases and other proteins likely to be involved in UV mutagenesis.

Pfeifer’s basic science findings are leading to a better understanding of the mechanisms of UV light and also lay the foundation for prevention and treatment of skin cancers.

## Figures and Tables

**Figure:**
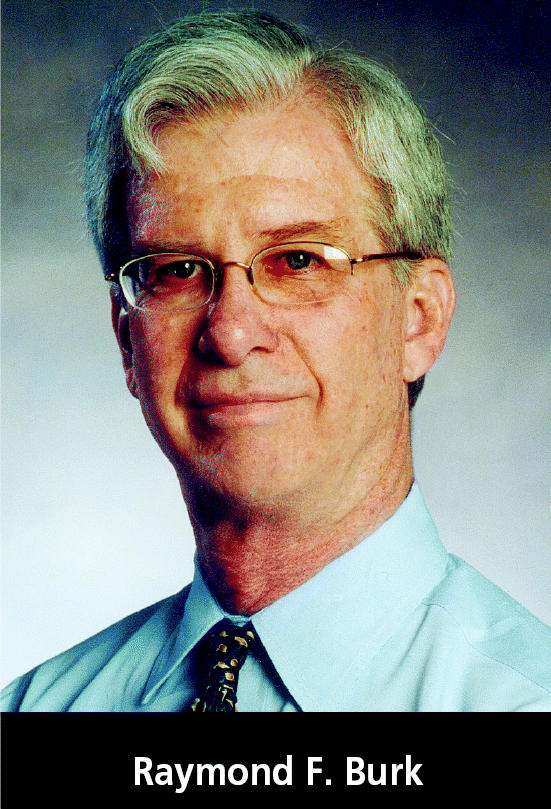


**Figure:**